# Hip fracture incidence in the elderly in Austria: An epidemiological study covering the years 1994 to 2006

**DOI:** 10.1186/1471-2318-8-35

**Published:** 2008-12-23

**Authors:** Eva Mann, Andrea Icks, Burkhard Haastert, Gabriele Meyer

**Affiliations:** 1 General practice and Institute for Health Services Research, Habsburgerstrasse 1, 6830 Rankweil, Austria; 2 Faculty of Public Health, Department of Epidemiology and International Public Health, School of Public Health, Bielefeld University, Bielefeld, Germany; and North-Rhine Westfalian Chamber of Physicians, Düsseldorf, Germany; 3 mediStatistica, Neuenrade, Germany; 4 Faculty of Medicine, Institute of Nursing Science, University Witten/Herdecke, Witten, Germany

## Abstract

**Background:**

Hip fractures in the elderly are a major public health burden. Data concerning secular trends of hip fracture incidence show divergent results for age, sex and regions. In Austria, the hip fracture incidence in the elderly population and trends have not been analysed yet.

**Methods:**

Hip fractures in the population of 50 years and above were identified from 1994 to 2006 using the national hospital discharge register. Crude incidences (IR) per 100,000 person years and standardised incidences related to the European population 2006 were analysed. Estimate of age-sex-adjusted changes was determined using Poisson regression (incidence rate ratios, IRRs).

**Results:**

The number of hospital admissions due to hip fracture increased from a total number of 11,694 in 1994 to 15,987 in 2006. Crude incidences rates (IR) per 100.000 for men increased from 244.3 (95% confidence interval (CI) 234.8 to 253.7) in 1994 to IR 330.8 (95% CI 320.8 to 340.9) in 2006 and for women from 637.3 (95% CI 624.2 to 650.4) in 1994 to IR 758.7 (95% CI 745.0 to 772.4) in 2006. After adjustment for age and sex the annual hip fracture incidence increase was only small but statistically significant (IRR per year 1.01, 95% CI 1.01 to 1.01, p < 0.01). Change of IRR over the 12 years study period was 13%. It was significantly higher for men (IRR over 12 years 1.21, 95% CI 1.16 to 1.27) than for women (IRR over 12 years 1.10, 95% CI 1.06 to 1.14) (interaction: p = 0.03).

**Conclusion:**

In contrast to findings in other countries there is no levelling-off or downward trend of hip fracture incidence from 1994 to 2006 in the Austrian elderly population. Further investigations should aim to evaluate the underlying causes in order to plan effective hip fracture reduction programmes.

## Background

Hip fractures in the elderly are a major public health problem throughout the industrialised Western societies. They are associated with individuals' increased morbidity [1] and functional impairment leading to care dependency and nursing home admission [2].

Recent studies show divergent results on time trends of hip fracture incidences in the elderly population. A Finnish study shows that the hip fracture incidence was stable between 1982 and 1993, but significantly increased in both sexes from 1992 to 2002 [3]. A recently published analysis of hip fracture trends in Germany indicates a decrease in women under the age of 74, but a significant increase in higher age [4]. Several studies show an increase of incidence rates for men and women [5,6]. However, a number of studies [7-11] show a levelling off or even a decline during the last decades.

In Austria, the nationwide incidence of hip fractures in the elderly population and its trend are unknown so far. Therefore, we aimed (1) to evaluate the incidence of hip fractures in the population of 50 years and older in Austria using the national hospital discharge diagnosis register, (2) to analyse the secular trend of hip fracture from 1994 to 2006, and (3) to evaluate differences in trends for sex and age groups.

## Methods

### Population and sample

Data from the national hospital discharge diagnosis register [12] were used. This register provides data on hospital discharges since 1989 and covers all hospitals in Austria.

The documentation is mandatory for all hospitals and is the basis for reimbursement.

Each hospital discharge is registered with date, age, sex, and residence of the patient, and diagnosis. Diagnoses are coded using the International Classification of Diseases (ICD). Hip fractures were counted by ICD 9 diagnosis 820 [13] up to the year 2000 and after 2000 by ICD 10 diagnosis S72.0, S72.1 and S72.2 [14].

European population data were taken from Eurostat database section on population and social conditions [15]. Austrian population data were provided from official statistics [16].

### Statistical Methods

Annual frequencies of hip fractures and corresponding incidences per 100,000 person years along with 95% confidence intervals (CIs) were calculated, overall and stratified for sex and age (strata: 50–59 years of age, 60–84 years of age 5 year strata, last stratum 85+), assuming Poisson distribution.

Standardised rates were estimated overall and stratified by sex using the European population in 2006 (27 countries) as standard population. To analyse the hip fracture incidence trends in Austria between 1994 and 2006, we used multiple Poisson regression models including age (same classes as above) and sex as confounders. Age-sex adjusted average annual changes (incidence rate ratios, IRRs) and changes over the whole study period of 12 years as average 12 years change IRR^12 ^were calculated. Two sided 95% CIs of IRRs were estimated based on the profile likelihood function. Furthermore, an interaction between sex and annual change was included in the Poisson model to test the difference of annual changes between men and women. To take into account overdispersion, all analyses were performed with DSCALE adjustment. The Poisson models were fitted based on count data stratified by state, year, sex-age class. These analyses were performed for the whole population (age ≥ 50 years) and stratified by sex and age-sex classes. The level of significance was 5%. All statistical tests were 2-sided. The Statistical Analysis Systems SAS (SAS for XP PRO, Release 9.2 TS1M0, SAS Institute Inc. Cary, NC, USA) was used for statistical analyses.

## Results

### Population

In the Austrian population the age group of 50 years and above increased from a total of 2,482,290 persons in 1994 to 2,816,168 in 2006, representing 31.3 and 34.1 percent of the total population [17]. The age groups of 80 to 84 and above 85 years, which represented 2.3% and 1.5% of the total Austrian population in 1994, increased to 2.7% and 1.7% in 2006. These two age groups represented 7.4% and 4.8% of the Austrian population aged 50 years and above in 1994 and 7.9% and 5.0% in 2006.

### Number and crude incidences of hip fractures

For the population aged 50 years and above the number of overall hip fractures increased from 11,694 in 1994 to 15,987 in 2006. Crude incidence rate per 100,000 person years (IR) increased from IR 471.1 (95% CI 462.6 to 479.6) in 1994 to IR 567.7 (95% CI 558.9 to 576.5) in 2006. For men IR increased from 244.3 (95% CI 234.8 to 253.7) in 1994 to IR 330.8 (95% CI 320.8 to 340.9) in 2006. For women IR increased from 637.3 (95% CI 624.2 to 650.4) in 1994 to IR 758.7 (95% CI 745.0 to 772.4) in 2006.

### Trend of hip fracture incidence

Additional file 1 displays the standardised incidences for the population of 50 years and above and stratified by sex between 1994 and 2006. Age- and sex-specific incidence rates are presented as well as average annual changes and changes over the whole study period from 1994 to 2006.

For the whole population at the age of 50 years and above multiple Poisson regression models showed a small but statistically significant average annual change of the hip fracture risk ratio of IRR 1.01 (95% CI 1.01–1.01, p < 0.01). Between 1994 and 2006 the change of overall IRR was 1.13 (95% CI 1.09–1.16).

However, annual changes and changes over the whole study periods differed markedly by age and gender. Increase over the whole study period was significantly higher in men (IRR 1.21, 95% CI 1.16 to 1.27) compared to women (IRR 1.10, 95% CI 1.06 to 1.14) (interaction p = 0.03).

Regarding age-strata, for men average annual changes of IRR per year showed a significant decrease for the age group 50 to 59 years and significant increases for the age group 65 to 69, 80 to 84 and 85 years and above. For women IRR change per year significantly increased in the age groups 80 to 84 years as well as 85 years and over. IRR over the whole study period seems to be highest for men at the age of 80 to 84 years and 85 years and above.

**Figure 1:**
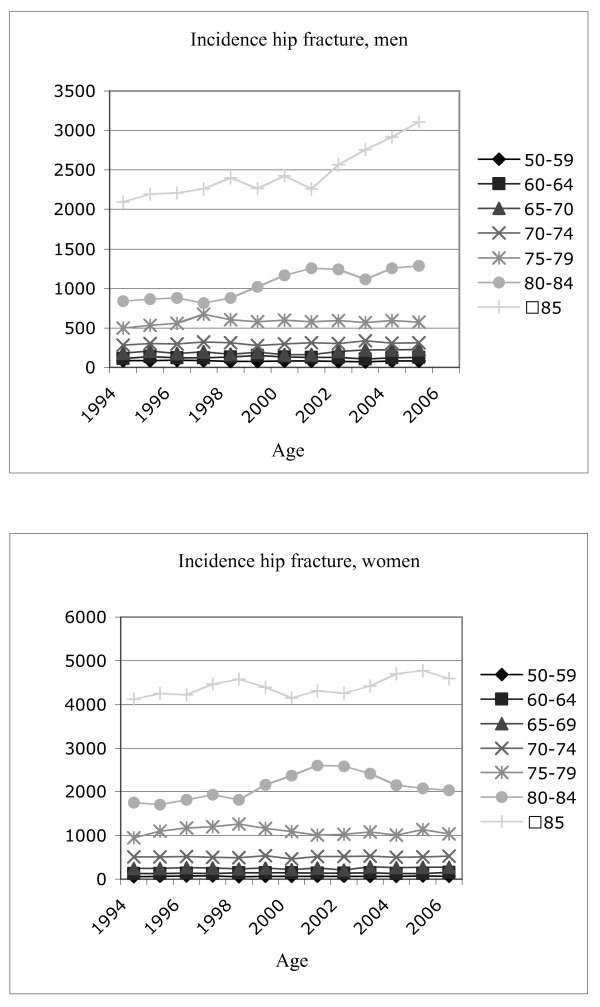
**Hip fracture incidence**.

Age- and sex-specific incidence rates from 1994 to 2006 are displayed in Figure 1.

## Discussion

This is the first study analysing incidence and secular trends of hip fracture in the population aged ≥ 50 years in Austria. In comparison to analyses from Germany [4], Spain [10] and Switzerland [8], the hip fracture incidence in Austria is higher, but it is lower than in Scandinavia [3].

Crude incidence of hip fractures increased between 1994 and 2006, mainly due to ageing of the population: After adjustment for age and sex the annual increase of hip fracture incidence was statistically significant but only small. Over the whole study period from 1994 to 2006 the incidence increase was found to be about 13%. Recent studies from other countries show different results concerning secular trends of hip fracture incidence. Whereas Lönmoos et al. in Finland [3] and Icks et al. in Germany [4] show an increase in hip fracture trend, Chevallery et al. in Switzerland [8] as well as Chang et al. in Australia [18] and Jaglal et al. in Canada [10] report a decrease.

Differences in hip fracture trends between men and women have been investigated in several studies. In Sweden, Löfman et al. [9] found a trend reversal in hip fracture incidence for women but not for men. In Denmark, a significant increase in hip fracture incidence was shown for men and women [19]. In Spain, Hernandez et al. [11] found that the crude IR increase occurred mainly to the disadvantage of women. In Germany, Icks et al. [4] found an incidence increase in men, a tendency of decrease in women up to the age of 74, and a pronounced increase in both, men and women, aged 74 years and above. In Switzerland, Chevalley et al. [8] reported a stable incidence of hip fractures for men and a significant decrease of incidence for women. However, our study revealed a statistically significant higher increase in hip fracture trend in men than in women.

Explanations for the overall and sex-and age adjusted increase and the pronounced increase of incidence of hip fractures in the oldest population can only be hypothesised.

One explanation of the high incidence rate of hip fractures in the oldest age groups might be the lack of a structured nationwide osteoporosis prevention approach [20], Furthermore, in Austria, no nationwide strategy on fall and fracture prevention has been implemented neither in nursing homes nor in community dwelling older people. However, this is also the case in most European countries. Nursing home residents represent a group of major concern, as the prevalence of hip fractures in this population is twofold up to threefold higher than in community dwelling elderly people [21]. Guillex et al. [22] showed, that the decrease in secular trend was attributable to the reduction of hip fractures in nursing homes. Due to data protection we could not determine the state of residence in our study.

Our study has several strengths. The Austrian hospital discharge diagnosis register covers all hospitals in Austria, using a continuous procedure since the introduction of the register. Our data cover all hip fractures collected through hospital diagnoses in Austria from 1994 to 2006.

Several limitations of our analysis have also to be considered. In contrast to other studies, a correction factor taking into account recurrent admissions and double registrations is not available for the Austrian hospital discharge register. As the main target of our analysis was the trend of the hip fracture incidences, results should be less affected.

We could not evaluate misclassification due to coding mistakes. However, a study in the UK found an excellent accuracy and reliability of hospital-coded records when compared to prospective hip fracture data collection [23]. Another possible shortcoming of our analysis might be the change in coding diagnoses of hip fractures from ICD 9 to ICD 10 in the year 2000, which could have led to coding mistakes. However, the diagnosis of a hip fracture is clearly described.

## Conclusion

The lack of a downward trend of hip fractures in the population aged ≥ 50 years in Austria emphasises the need of interventions and continuous monitoring of the hip fracture incidence. Further investigations should aim to analyse the underlying causes for hip fractures and compare our results with the results of countries, which show a levelling off or even a decline in hip fracture trend. Such information would be a necessary basis for future structured and nationwide interventions to achieve a meaningful hip fracture reduction.

## Competing interests

The authors declare that they have no competing interests.

## Authors' contributions

EM, AI and GM initiated the study. EM and GM developed the study protocol. EM coordinated the data analysis, and interpreted the results with help of the other authors. BH performed the statistical analysis. EM wrote the paper. All authors commented on paper drafts. EM is guarantor for the paper.

## Pre-publication history

The pre-publication history for this paper can be accessed here:



## Supplementary Material

Additional file 1 **Additional table**. Incidence rates of hip fractures and trends in Austria (1994 to 2006). Click here for file
