# Supplementary material for: Hip fracture incidence in the elderly in Austria: An epidemiological study covering the years 1994 to 2006
Source: BMC Geriatr. 2008 Dec 23;8:35. doi: 10.1186/1471-2318-8-35 (PMC2629766; doi:10.1186/1471-2318-8-35)
Supplement: Additional file 1 — Additional table. Incidence rates of hip fractures and trends in Austria (1994 to 2006). [file 1471-2318-8-35-S1.doc]

**Incidence rates of hip fractures and trends in Austria (1994 to 2006).**

| 1994 1995 1996 1997 1998 1999 2000 2001 2002 2003 2004 2005 2006 IRR per year IRR^12^ 1994-2006 |
| --- |
| P^*^ > 50 (m) 2,482 2,505 2,504 2,520 2,553 2,586 2,616 2,645 2,682 2,708 2,736 2,773 2,816 |
| n^†^ HF 11,694 12,394 12,891 13,484 13,603 13,924 13,774 14,433 14,682 15,131 14,893 15,698 15,987 |
| **______________________________________________________________________________________________________________________________________________** |
|  |
| **Total**^‡^ 473.5 495.8 508.0 526.5 521.5 532.5 524.6 543.2 538.7 551.9 541.9 558.5 550.8 1.01^**^ 1.13 (1.09-1.16) |
| **Men**^§^ 315.6 340.1 339.6 353.5 348.9 352.0 359.5 368.7 366.0 385.0 386.4 407.9 420.5 1.02^**^ 1.21 (1.16-1.27) |
| **Woman**^§^ 557.3 577.9 600.0 617.5 614.4 630.3 612.6 637.4 630.7 643.0 624.7 640.1 622.8 1.01^**^ 1.10 (1.06-1.14) |
|  |
| **Male, age** |
| 50-59 87.5 84.6 91.0 87.4 73.4 73.4 83.1 78.5 78.0 74.6 72.9 80.2 79.6 0.99^*^ 0.88 (0.78-0.99) |
| 60-64 109.9 133.8 125.2 121.7 131.7 153.2 139.6 130.8 127.8 126.5 106.8 120.6 126.2 1.00 0.96 (0.83-1.10) |
| 65-69 181.9 208.2 174.0 195.8 167.0 191.0 142.6 160.1 161.4 203.1 220.1 222.4 228.5 1.02^**^ 1.21 (1.06-1.39) |
| 70-74 279.9 303.0 296.1 323.1 311.1 277.7 340.0 296.1 311.0 300.4 337.1 305.2 311.9 1.01 1.07 (0.94-1.21) |
| 75-79 499.0 533.0 558.7 674.4 602.9 576.5 545.3 597.1 576.0 593.4 565.8 591.7 575.1 1.01 1.07 (0.95-1.20) |
| 80-84 837.5 866.0 877.6 812.8 878.7 1023.0 1151.3 1167.6 1255.5 1241.6 1116.1 1256.8 1289.6 1.04^**^ 1.61 (1.44-1.80) |
| ≥ 85 2090.0 2194.2 2207.5 2257.8 2399.9 2261.0 2247.0 2421.8 2259.3 2562.2 2757.3 2914.9 3108.2 1.03^**^ 1.42 (1.26-1.59) |
|  |
| **Female, age** |
| 50-59 58.7 61.4 70.2 69.0 58.9 61.4 62.7 59.6 66.0 59.9 64.4 69.3 64.5 1.00 1.04 (0.91-1.19) |
| 60-64 123.4 127.6 138.2 124.7 142.0 148.2 141.4 138.5 136.0 145.1 123.4 126.3 154.4 1.01 1.07 (0.93-1.23) |
| 65-69 244.5 246.8 260.9 251.7 231.3 251.5 221.2 252.7 205.2 278.1 265.0 272.5 269.1 1.01 1.09 (0.98-1.20) |
| 70-74 510.0 510.5 521.1 500.8 491.3 532.5 466.3 517.8 512.7 524.2 502.7 508.0 528.0 1.00 1.01 (0.93-1.10) |
| 75-80 940.1 1095.1 1169.9 1200.3 1256.1 1163.7 1087.6 1002.6 1023.3 1078.6 1004.0 1128.4 1035.6 0.99 0.93 (0.84-1.02) |
| 80-84 1746.4 1700.8 1809.5 1930.6 1809.1 2154.0 2367.5 2596.7 2580.2 2414.1 2145.9 2076.2 2029.1 1.02^**^ 1.27 (1.15-1.40) |
| ≥ 85 4122.6 4249.5 4227.3 4472.2 4574.2 4398.6 4148.5 4311.4 4263.5 4421.8 4702.2 4780.9 4599.6 1.01^*^ 1.10 (1.02-1.19) |
|  |

^*^Population at the age of 50 years and above (P > 50), number in million (m). ^†^Number of hip fractures (n HF).

^‡^Standardised to the European population 2006. ^§^Age-standardised incidence rates per 100,000 (95% confidence interval).

IRR: Incidence rate ratio (95% confidence interval), from age-sex-adjusted Poisson regression. ^*^p < 0.05 ^**^p < 0.01.
